# Declining Artesunate-Mefloquine Efficacy against Falciparum Malaria on the Cambodia–Thailand Border

**DOI:** 10.3201/eid1405.071601

**Published:** 2008-05

**Authors:** Chansuda Wongsrichanalai, Steven R. Meshnick

**Affiliations:** * US Naval Medical Research Unit No. 2, Jakarta, Indonesia; † University of North Carolina at Chapel Hill School of Public Health, Chapel Hill, North Carolina, USA

**Keywords:** malaria, artesunate-mefloquine, drug resistance, Cambodia, Thailand, pfmdr1, Plasmodium falciparum, ACT, anti-malarial, rational therapy, synopsis

## Abstract

Resistance to many antimalaria drugs developed on the Cambodia–Thailand border long before developing elsewhere. Because antimalaria resistance is now a global problem, artemisinin-based combination therapies (ACTs) are the first-line therapies in most malaria-endemic countries. However, recent clinical and molecular studies suggest the emergence of ACT-resistant *Plasmodium falciparum* infections in the Cambodia–Thailand border area, where standard ACT is artesunate and mefloquine. These ACT failures might be caused by high-level mefloquine resistance because mefloquine was used for monotherapy long before the introduction of ACT. This observation raises 2 questions. First, how can existing *P. falciparum–*resistant strains be controlled? Second, how can the evolution of new ACT- resistant strains be avoided elsewhere, e.g., in Africa? Enforcement of rational drug use and improved diagnostic capacity are among the measures needed to avoid and contain ACT resistance.

## Abstract 2

Emerging resistance in Southeast Asia raises concern over possible spread or similar evolution of resistance to other artemisinin-based combination therapies in Africa.

Artemisinin-based combination therapies (ACTs), considered the best current treatment for falciparum malaria (*1*), have energized worldwide programs to control malaria. Combination therapies, in general, tend to delay the development of microbial resistance. However, several ACT regimens are combinations of artesunate and older antimalarial drugs against which resistance already exists. Preexisting resistance to these older partner drugs could lead to drug failure. This may have already happened on the Cambodia–Thailand border (*2*).

## Historical Perspective

The western Cambodia–southeastern Thailand border, which comprises the areas around the town of Pailin in Cambodia and the provinces of Trat and Chanthaburi in Thailand, has been an epicenter of drug-resistant malaria (*3*) (Figure). Resistance to chloroquine and sulfadoxine-pyrimethamine occurred there in the late 1950s and 1960s, respectively. Mefloquine was introduced there in 1983, first on the Thai side and in the form of mefloquine in combination with sulfadoxine-pyrimethamine. Mefloquine resistance led to replacement by artesunate-mefloquine in Thailand in 1995. In Chanthaburi and Trat Provinces, considered by the Thai national malaria control program to be the areas with the highest level of mefloquine resistance, the dosages were 12 mg/kg artesunate and 25 mg/kg mefloquine or a maximum adult dose of 600 mg artesunate and 1,250 mg mefloquine, given for 2 days. This regimen was 99% efficacious when field tested in the same border areas in 1993 (*4*). It also ensured better compliance than an extended (3-day) regimen. With the exception of the southeastern border of Thailand with Cambodia, the regimen remained effective throughout Thailand even when the control program switched to a 3-day treatment course in 2007 in accordance with the World Health Organization (WHO) recommendation.

**Figure:**
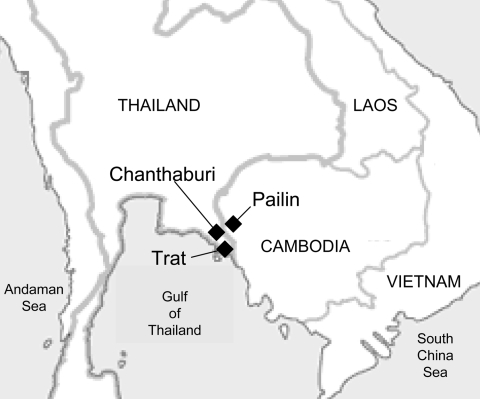
Map of the Cambodia–Thailand border showing the town of Pailin, Cambodia, and the provinces of Chanthaburi and Trat, Thailand; the areas are collectively known as the epicenter of drug-resistant malaria.

In Cambodia in 2000, artesunate-mefloquine became the first-line drug for the treatment of falciparum malaria. The dosages were 12 mg/kg artesunate and 20 mg/kg mefloquine, or a maximum adult dose of 600 mg artesunate and 1,000 mg mefloquine, given for 3 days, in accordance with the WHO recommendation. The lower mefloquine dose was based on the perception that Cambodians were slightly smaller than Thais in body build. Malaria control programs of each country regularly monitor antimalarial therapeutic efficacy at selected sentinel sites.

### Resistance to Artesunate-Mefloquine

Emerging resistance is supported by 3 independently conducted studies. In Pailin in 2002, clinical monitoring of response to combination artesunate (≈12 mg/kg) and mefloquine (≈20 mg/kg) co-blister packs (artesunate for 3 days and mefloquine for 1 day) showed 85.7% efficacy at day 28 follow-up (*5*) (Table). A repeat study in Pailin in 2004, which used the same drug combination but more precise dosing and follow-up at 42 days, found efficacy to be 79.3% (*5*). To exclude cases of reinfection from analysis, parasite variants were identified by using nested PCR amplification of 3 polymorphic genes for merozoite surface protein 1 (*msp1*), *msp2*, and glutamate-rich protein (*7*). In Thailand’s Trat Province in 2003, efficacy of 78.6% (95% confidence interval 66.4%–91.1%) was reported from a 28-day follow-up study of 44 patients who received the same total dosage of this combination in a 2-day regimen (*6*).

**Table:** Studies that demonstrated poor artesunate-mefloquine efficacy, Cambodia–Thailand border*

Reference	Study site, country, y	ACT	No. patients	Follow-up duration, d	Efficacy, %
Denis et al., 2006 (*5*)	Pailin, Cambodia, 2002	ATS ≈12 mg/kg in 2 doses on days 0, 1, and 2 + MFQ ≈20 mg/kg in 2 doses on day 0	70 children and adults	28	85.7 (PCR-corrected)
Vijaykadga et al., 2006 (*6*)	Trat, Thailand, 2003	ATS 12 mg/kg (maximum 600 mg) in 2 doses on days 0 and 1 + MFQ 25 mg/kg (maximum 1,250 mg) in 2 doses on day 0	44, age >10 y, mostly adults	28	78.6
Denis et al., 2006 (*5*)†	Pailin, Cambodia, 2004	ATS 12 mg/kg in 2 doses on days 0, 1, and 2 + MFQ 25 mg/kg in 2 doses on day 0	58 children and adults	42	79.3 (PCR-corrected)

*ACT, artemisinin-based combination therapy; ATS, artesunate; MFQ, mefloquine; day 0, first 24 h of enrollment and start of therapy. †Also in this study, increased copy numbers of *Plasmodium falciparum* multidrug resistance 1 gene were found to be associated with parasite recrudescence, and as many as 44% of patients did not clear parasites until after 48 hours.

Each of the 3 studies used directly observed therapy, followed the WHO standard in vivo study protocol, and obtained from their respective national control programs reliable drugs that worked effectively at other sentinel sites. Although each of the 3 studies was small, in aggregate they attest to a worrisome level of in vivo artesunate-mefloquine resistance.

The above clinical observations are supported by molecular evidence. High copy numbers of the *Plasmodium falciparum* multidrug resistance 1 (*pfmdr1*) gene, a marker of multidrug resistance (*8*), predicted recrudescence in the 2004 Pailin study, even after PCR correction and adjustment for age and parasite density (*9*). Thus, clinical and molecular evidence indicate that artesunate-mefloquine failures are occurring on the Cambodia–Thailand border. In contrast, artesunate-mefloquine remains effective in eastern Cambodia and elsewhere in Thailand.

In field situations, artesunate-mefloquine failure is likely to be worse than in controlled studies because of poorer compliance and variations in drug quality. Some of the results presented above might have overestimated artesunate-mefloquine therapeutic efficacy because 1) 28-day follow-up is inadequate time to evaluate drugs with a long elimination half-life, such as mefloquine for which up to 60% of recrudescence may occur between day 28 and day 42 (*9*), and 2) the current genotyping method is likely to misclassify some recrudescence as reinfection because of the method’s limited ability to detect minor subpopulations of parasites that carry drug resistance mutations (J.J. Juliano, pers. comm.). In our opinion, PCR correction to classify recurrent infections is not necessary for a low-transmission area such as the Cambodia–Thailand border, where infection frequently results from occupational exposure in the jungles and patients usually remain at low risk for reinfection while in the village during study follow-up.

These treatment failures most likely result from mefloquine resistance rather than artemisinin resistance because increased *pfmdr1* copy number has been linked most closely with mefloquine resistance in vivo (*10**,**11*). Monitoring of in vitro antimalaria drug susceptibility by the Pasteur Institute of Cambodia since 2001 also suggests progressive loss of mefloquine sensitivity in western Cambodia (*2*). Most likely, mefloquine resistance in this area had already reached a level too extreme for the drug to be further protected by artesunate. To some extent, the development of resistance to artesunate-mefloquine in areas of preexisting mefloquine resistance mirrors the experience with the mefloquine-sulfadoxine-pyrimethamine combination that was introduced to deal with widespread preexisting sulfadoxine-pyrimethamine resistance (*3*).

Fortunately, no clear evidence points to artesunate failure, although it is possible. No mutations thought to be associated with artemisinin resistance have been detected in Cambodia, and no isolates resistant to artesunate in vitro have been found (*12*). However, the increased parasite clearance times recorded in Pailin are worrisome. In the Pailin 2004 study, as many as 34% of patients cleared parasites between 48 and 72 hours, and 10% after 72 hours (*9*). When artesunate was first introduced in the early 1990s, failure to clear parasites by 48 hours was rare among patients with uncomplicated falciparum malaria in southeastern Thailand. Additionally, increased copy numbers of *pfmdr1* gene have recently been shown to be associated with in vitro resistance to artemisinin (and to lumefantrine and quinine) (*13*).

## Likely Resistance Factors

Several reasons may explain the emergence of artesunate-mefloquine resistance on the Cambodia–Thailand border. First, the concept of rational therapy is poorly reinforced. Improper use of antimalaria drugs based on clinical diagnosis alone or on misdiagnosis as a result of poor microscopy technique or interpretation could have accelerated the onset of resistance. In Cambodia, because of poor transportation and public health infrastructure, artesunate and mefloquine are made available in the private sector to increase patients’ access to the drug. This access, in turn, increases the risks that drug quality will be substandard and drug use will be uncontrolled (*5*). Because adherence and indication are not adequately emphasized, drugs are consumed in incomplete dosages or for prophylaxis such as before a jungle trip. Social marketing helps to control drug quality but cannot ensure adherence.

Although ACT use has been restricted and prescription is based on microscopic confirmation at government-run malaria clinics in Thailand, ACT is less well-controlled in Cambodia. Unreliable services and poor diagnostic capabilities at peripheral health facilities further discourage patients from seeking malaria treatment from the public sector and encourage self-purchase of drugs. A recent study of malaria treatment–seeking behavior in Cambodia showed that >80% of the patients initially sought treatment from private providers and pharmacies or consumed drugs on their own (*14*).

Second, the short half-life of artesunate relative to that of mefloquine means that tolerance to mefloquine could develop when treated patients are reinfected (*15*). Third, the malaria parasites in this region could have a unique ability to develop resistance to any antimalaria drugs (*16*); their genetics need to be further studied.

## Possible Development of New Foci of Resistance

As of 2007, reduced efficacy of artesunate-mefloquine was noted in Kampot, a province southwest of Phnom Penh (*17*). Thus, resistant parasites may be spreading. Although no other ACT is immediately ready for field use, the Greater Mekong subregion will soon need alternatives to artesunate-mefloquine.

In sub-Saharan Africa, presumptive treatment with ACT may soon become the norm; this drug-use practice may similarly promote evolution of resistance. ACT resistance in Africa could be devastating. This concern has been raised repeatedly (*18**,**19*). Although artesunate-mefloquine is not recommended for African countries, 1 of the ACTs now used is the combination of artemether and lumefantrine (Coartem; Novartis, Basel, Switzerland); lumefantrine is chemically related to mefloquine. One way to delay the emergence of resistance would be to enforce ACT prescription based on accurate biological diagnosis.

## Conclusions

Studies are now under way to replicate these initial findings on the Cambodia–Thailand border (*20*). Nevertheless, existing data strongly suggest that artesunate-mefloquine resistance exists in this area. This finding is a warning message for Africa, where ACT has been used in a large scale but not with parallel effort to enhance rational therapy. Continued surveillance for ACT resistance should be an integral part of any malaria control program that uses these drugs.

## References

[1] World Health Organization. Guidelines for the treatment of malaria. WHO/HTM/MAL 2006.1108 [cited 2008 Mar 5]. Geneva: The Organization; 2006. Available from http://www.who.int/malaria/docs/TreatmentGuidelines2006.pdf

[2] World Health Organization. Containment of malaria multi-drug resistance on the Cambodia-Thailand border. Report of an informal consultation, Phnom Penh, 29–30 January 2007. Report no. SEA-MAL-246 [cited 2008 Mar 5]. Available from http://www.who.int/malaria/docs/drugresistance/ReportThaiCam.pdf

[3] Wongsrichanalai C, Prajakwong S, Meshnick SR, Shanks GD, Thimasarn K. Mefloquine—its 20 years in the Thai Malaria Control Program. Southeast Asian J Trop Med Public Health. 2004;35:300–8. 15691128

[4] Thimasarn K, Sirichaisinthop J, Chanyakhun P, Palananth C, Rooney W. A comparative study of artesunate and artemether in combination with mefloquine on multidrug resistant falciparum malaria in eastern Thailand. Southeast Asian J Trop Med Public Health. 1997;28:465–71. 9561593

[5] Denis MB, Tsuyuoka R, Poravuth Y, Narann TS, Seila S, Lim C, Surveillance of the efficacy of artesunate and mefloquine combination for the treatment of uncomplicated falciparum malaria in Cambodia. Trop Med Int Health. 2006;11:1360–6. 10.1111/j.1365-3156.2006.01690.x 16930257

[6] Vijaykadga S, Rojanawatsirivej C, Cholpol S, Phoungmanee D, Nakavej A, Wongsrichanalai C. In vivo sensitivity monitoring of mefloquine monotherapy and artesunate-mefloquine combinations for the treatment of uncomplicated falciparum malaria in Thailand in 2003. Trop Med Int Health. 2006;11:211–9. 10.1111/j.1365-3156.2005.01557.x 16451346

[7] Snounou G, Beck HP. The use of PCR genotyping in the assessment of recrudescence or reinfection after antimalarial drug treatment. Parasitol Today. 1998;14:462–7. 10.1016/S0169-4758(98)01340-4 17040849

[8] Cowman MB, Saliba KJ, Caruana SR, Kirk K, Cowman AF. Pgh1 modulates sensitivity and resistance to multiple antimalarials in *Plasmodium falciparum.* Nature. 2000;403:906–9. 10.1038/35002615 10706290

[9] Alker AP, Lim P, Sem R, Shah NK, Yi P, Bouth DM, *pfmdr1* and in vivo resistance to artesunate-mefloquine in falciparum malaria on the Cambodian–Thai border. Am J Trop Med Hyg. 2007;76:641–7. 17426163

[10] Nelson AL, Purfield A, McDaniel P, Uthaimongkol N, Buathong N, Sriwichai S, *pfmdr1* genotyping and in vivo mefloquine resistance on the Thai-Myanmar border. Am J Trop Med Hyg. 2005;72:586–92. 15891133

[11] Price RN, Uhlemann AC, Brockman A, McGready R, Ashley E, Phaipun L, Mefloquine resistance in *Plasmodium falciparum* and increased *pfmdr1* gene copy number. Lancet. 2004;364:438–47. 10.1016/S0140-6736(04)16767-6 15288742 PMC4337987

[12] Jambou R, Legrand E, Niang M, Khim N, Lim P, Volney B, Resistance of *Plasmodium falciparum* field isolates to in-vitro artemether and point mutations of the SERCA-type *PfATPase6.* Lancet. 2005;366:1960–3. 10.1016/S0140-6736(05)67787-2 16325698

[13] Sidhu AB, Uhlemann AC, Valderramos SG, Valderramos JC, Krishna S, Fidock DA. Decreasing *pfmdr1* copy number in *Plasmodium falciparum* malaria heightens susceptibility to mefloquine, lumefantrine, halofantrine, quinine, and artemisinin. J Infect Dis. 2006;194:528–35. 10.1086/507115 16845638 PMC2978021

[14] Duong S, Chanthap L, Babu VVRS. Piloting of a strategy for collection of malaria information from the private sector, Cambodia, Final Report, March 2005. National Centre for Parasitology, Entomology and Malaria Control GTZ BACKUP Initiative. Phnom Penh: The Initiative; 2005

[15] Hastings IM, Ward SA. Coartem (artemether-lumefantrine) in Africa: the beginning of the end? J Infect Dis. 2005;192:1303–4. 10.1086/432554 16136476

[16] Rathod PK, McErlean T, Lee PC. Variations in frequencies of drug resistance in *Plasmodium falciparum.* Proc Natl Acad Sci U S A. 1997;94:9389–93. 10.1073/pnas.94.17.9389 9256492 PMC23200

[17] Sem R, Lim P, Muth S, Kim S, Chim P, Duong S, Efficacy of current standard therapy for uncomplicated *P. falciparum* and *P. vivax* malaria in central Cambodia. Abstracts of the Joint International Tropical Medicine Meeting 2007 “Health Security in the Tropics"; 2007 Nov 29–30; Bangkok. Abstract No. 47.

[18] Wongsrichanalai C, Thimasarn K, Sirichaisinthop J. Antimalarial drug combination policy: a caveat. Lancet. 2000;355:2245–7. 10.1016/S0140-6736(00)02416-8 10881909

[19] Bloland PB, Ettling M, Meek S. Combination therapy for malaria in Africa: hype or hope? Bull World Health Organ. 2000;78:1378–88. 11196485 PMC2560651

[20] World Health Organization. Resistance to artemisinin derivatives along the Thai-Cambodian border. Wkly Epidemiol Rec. 2007;82:360. 17933087

